# Effects of Controlled-Release Fertilizer on Leaf Area Index and Fruit Yield in High-Density Soilless Tomato Culture Using Low Node-Order Pinching

**DOI:** 10.1371/journal.pone.0113074

**Published:** 2014-11-17

**Authors:** Takafumi Kinoshita, Takayoshi Yano, Makoto Sugiura, Yuji Nagasaki

**Affiliations:** 1 NARO Tohoku Agricultural Research Center, National Agriculture and Food Research Organization (NARO), Morioka, Iwate, Japan; 2 NARO Western Region Agricultural Research Center, National Agriculture and Food Research Organization (NARO), Zentsuji, Kagawa, Japan; Tennessee State University, United States of America

## Abstract

To further development of a simplified fertigation system using controlled-release fertilizers (CRF), we investigated the effects of differing levels of fertilizers and plant density on leaf area index (LAI), fruit yields, and nutrient use in soilless tomato cultures with low node-order pinching and high plant density during spring-summer (SS), summer-fall (SF), and fall-winter (FW) seasons. Plants were treated with 1 of 3 levels of CRF in a closed system, or with liquid fertilizer (LF) with constant electrical conductivity (EC) in a drip-draining system. Two plant densities were examined for each fertilizer treatment. In CRF treatments, LAI at pinching increased linearly with increasing nutrient supply for all cropping seasons. In SS, both light interception by plant canopy at pinching and total marketable fruit yield increased linearly with increasing LAI up to 6 m^2^·m^−2^; the maximization point was not reached for any of the treatments. In FW, both light interception and yield were maximized at an LAI of approximately 4. These results suggest that maximizing the LAI in SS and FW to the saturation point for light interception is important for increasing yield. In SF, however, the yield maximized at an LAI of approximately 3, although the light interception linearly increased with increasing LAI, up to 4.5. According to our results, the optimal LAI at pinching may be 6 in SS, 3 in SF, and 4 in FW. In comparing LAI values with similar fruit yield, we found that nutrient supply was 32−46% lower with the CRF method than with LF. In conclusion, CRF application in a closed system enables growers to achieve a desirable LAI to maximize fruit yield with a regulated amount of nutrient supply per unit area. Further, the CRF method greatly reduced nutrient use without decreasing fruit yield at similar LAIs, as compared to the LF method.

## Introduction

Development of a simplified, low-cost, and high-yield system in soilless agriculture is very important, particularly for small-scale growers. Compared to liquid fertilizer (LF), which is commonly used in soilless cultures, controlled-release fertilizer (CRF) is simple and economical because it does not require equipment to adjust nutrient concentration and fertilizer delivery.

Efficient nutrient use in a soilless culture is necessary for both economic and environmental reasons. Normally, plants are supplied with a nutrient solution with a constant electrical conductivity (EC) (EC-based control). The EC value for each growth stage is determined empirically, and the amount of nutrients supplied typically exceeds the plants' demand. As there is currently no regulation for the control of farm effluents in Japan, unused nutrient solution is often discarded. Such inefficient use of nutrients is wasteful and costly for growers. Even when surplus nutrients are recycled, periodic nutrient composition analysis is required to maintain the nutrient balance, forcing growers to depend on commercial analytical services. Therefore, introducing a water-recirculating (closed) system is often cost prohibitive, particularly for small growers.

An alternative nutrient supply procedure called quantitative nutrient management (QNM) was recently proposed as a more efficient way to use inorganic nutrients in soilless cultures of tomato [1]–[3] and melon [4]. In QNM, the nutrients required for plants are released at a set period without further additional nutrient supply, and it is therefore possible to suppress excess nutrient absorption, and to cultivate crops in a closed system with less wasteful nutrient accumulation in the water-circulation tank. Using QNM in a closed system reduces nutrient supply to a greater extent than with an EC-based control in a drip-draining system, but without reducing the fruit yield [1], [5], [6].

The application of CRF in a soilless culture is considered to be QNM because it enables growers to supply nutrients according to the specific requirements of the plants without excess nutrient supply. Therefore, application of CRF in a closed system may also improve nutrient-use efficiency, reducing the cost of introducing a closed system compared to LF application with an EC-based control in a habitual drip-draining system. In addition, applying CRF to a water-circulating tank rather than to substrate simplifies fertilizer application.

In our previous study, we demonstrated that application of CRF to a water-circulating tank in a root-proof capillary wick irrigation system, a type of sub-irrigation method, resulted in the same quantity of fruit production with high nutrient-utilization efficiency through the suppression of excess nutrient uptake, as compared to LF application [7], [8]. Moreover, we reported that application of CRF to a water circulating tank in a closed system also greatly reduced nutrient supply without reducing fruit yield, compared to LF- and EC-based management used in an open water-draining system [9].

Controlling the leaf area index (LAI) is also very important for high-yield tomato production. The fraction of light intercepted by the tomato canopy shows a positive, saturating-type response to increased LAI; intercepted light increases with the increasing LAI until 3−4 m^2^·m^−2^ but any further increase in LAI has only a marginal effect on canopy light interception [10]. Fruit yields similarly respond to LAI [11], [6], reflecting the linear relationship between fruit yields and solar radiation on tomato crops [12]. The optimal LAI for yields differs depending on the growing season [11], mainly due to changes in solar radiation. This indicates the importance of controlling LAI suitably according to the season.

In QNM, it may be possible to control LAI by adjusting nutrient supply and plant density by lowering nutrient use, as compared to levels in EC-based management at similar LAI values [6]. In our previous study, such results were obtained when CRF was applied to a closed system [9]. Therefore, it is probably possible to control optimal LAI, depending on the growing season, by using CRF in a closed system.

Recently, a tomato cultivation method with a low node-order pinching system and high plant density has become a practical and widely used technique for year-round production and increased yields in Japan [13]. This method is characterized by the following: (1) plant density is high with harvesting of 1 to 4 trusses, (2) cultivation period is short (commonly 70−120 days from transplant to the end of harvest), (3) it is continuously repeated to attain year-round cultivation. Plant height is comparably low with this method, making it acceptable for small-scale growers because the height of their greenhouses is also comparably low.

With this background, we investigated the effects of differing amounts of CRF application and plant density on LAI, fruit yield, and nutrient-use efficiency in a soilless tomato culture with a low node-order pinching system and high plant density in 3 different growing seasons. Using our data, we also evaluated the optimal fertilizer management in relation to LAI.

## Materials and Methods

### Ethics statement

1. The NARO Western Region Agricultural Research Center (lat. 34°13′N, long. 133°46′E) is the experimental base of the National Agriculture and Food Research Organization (NARO). Therefore, the authority that issued the permit for this location is NARO.
2. No specific permissions were required for this location. The field studies did not involve endangered or protected species.

### Plant material and growth conditions

**Figure 1:**
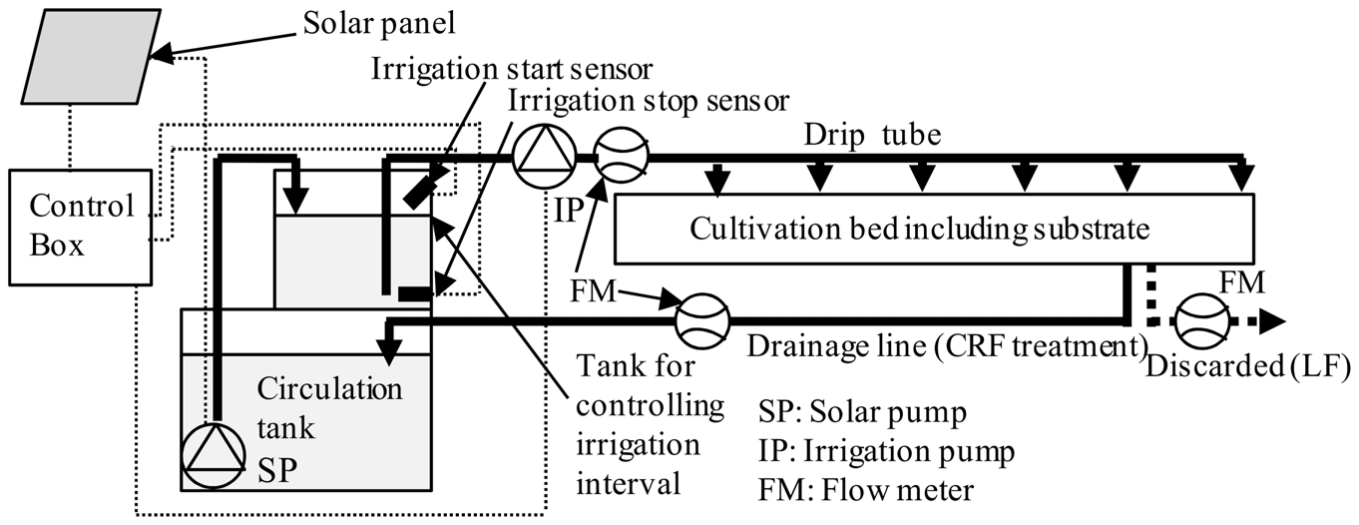
Diagram of the soilless culturing system used in this study. Irrigation starts when the level of nutrient solution reaches the irrigation start sensor in the tank, which controls the irrigation interval, by operating the solar pump. Irrigation stops when the level of nutrient solution decreases to the level of the irrigation stop sensor. The cycle repeats as necessary. Since the flow rate of the solar pump depends on solar power, so does irrigation frequency.

The experiment was conducted 3 times, during the spring-summer (SS), summer-fall (SF), and fall-winter (FW) seasons in a plastic greenhouse (area  = 108 m^2^) at the NARO Western Region Agricultural Research Center, Zentsuji, Kagawa, Japan (lat. 34°13′N, long. 133°46′E). Seeds of tomato plants with large fruits (‘Momotaro Fight’, Takii Seed Co., Kyoto, Japan) were sown in 128-well plug trays filled with commercial growth medium (Yosaku N150; JA Zen-noh, Tokyo, Japan) on March 8, 2012 (SS); July 23, 2012 (SF); and October 2, 2012 (FW). Seedlings were transferred to 9-cm polyethylene pots filled with a mixed substrate (mountain sand:bark compost:perlite:peatmoss:vermiculite  = 5:9:8:2:2 [v/v]) on April 2, 2012 (SS); July 11, 2012 (SF); and October 16, 2012 (FW). On April 20, 2012 (SS); July 27. 2012 (SF); and November 11, 2012 (FW), the seedlings, just before flowering of the first truss, were transplanted to a plastic bed (300 cm long ×28 cm wide ×14 cm high) filled with the same substrates described previously, to form a double line of plants (with a distance between lines of 20 cm) at different plant densities, as described in the following section (Figure 1). Each plant was irrigated from the circulation tank 1−8 times per day using a drip tube (0.5 L·h^−1^ per plant, Hydrogol 12/35/1; Plastro, Israel) with a solar-mediated system operated by an automated pulsating drip-irrigation system, driven by a solar pump [14]. The irrigation rate was approximately 200 mL per plant per irrigation event. Plants were topped, leaving 2 leaves above the 4th truss, and harvesting continued until July 20, 2012 (SS); October 24, 2012 (SF); and March 21, 2013 (FW). The beds were arranged in rows from south to north, and the distance between the beds was 1.8 m. As the plants grew, all lateral shoots were removed, and the remaining single stem was trained vertically on a string attached to a horizontal wire at a height of 2.2 m. The flowering trusses were treated with 15 mg·L^−1^ of *p*-chlorophenoxyacetic acid (4-CPA) to promote fruit set. Trusses were thinned to contain no more than 5 fruits. The greenhouse was heated at night to maintain a minimum temperature of 13°C, and ventilation was initiated during the daytime when the temperature was higher than 25°C. Carbon dioxide enrichment and artificial lighting were not used in the greenhouse.

### Treatments

Plants were grown in 1 of 4 fertilizing treatments (Table 1): CRF-L (low), CRF-M (medium), CRF-H (high) or LF. For the CRF treatments, plants were supplied with nutrients in a closed system, where all drainage from the bed returned to the circulation tank (50 L). Nutrient levels for CRF-H were 3 times and 1.5 times higher than CRF-L or CRF-M, respectively. All CRFs were supplied in the circulation tank on the day of seedling planting. In the LF treatment, nutrient solution was irrigated with an EC control (the EC was adjusted to 1.4 dS•m^−1^, which is a conventional value in Japan) in a drip-draining system, where all drainage from the bed was discarded. In the LF treatment, the average efflux percentage over inflow nutrient solution during the experiment was approximately 33% (SS) and 30% (SF and FW), which is standard level for Japanese soilless cultures.

**Table 1:** Nutrient components (g/plant) of fertilizers added to the water tank for cultivation of large-fruited tomato.

Treatment^a^	Japanese standard name of fertilizer	Days^b^		N	P	K_2_O	CaO	MgO	Fe	Mn	Zn	Cu	B	Mo
		SS and SF	FW	(g/plant)					(mg/plant)					
CRF-L	Eco long total 313^c^	100	70	0.5	0.2	0.5		0.1	8.0	4.0	0.6	2.0	2.4	0.8
	Super eco long^d^	s100	s70	0.6	0.2	0.6								
	Long calcium nitrate	100	100	1.2			2.4							
	Coating potassium	100	100	0.2		3.8								
	Long magnesium sulfate	100	100					0.5						
	Total			2.5	0.4	4.9	2.4	0.6	8.0	4.0	0.6	2.0	2.4	0.8
CRF-M	Eco long total 313	100	70	1.0	0.4	1.0		0.2	16.0	8.0	1.2	4.0	4.8	1.6
	Super eco long	s100	s70	1.1	0.4	1.1								
	Long calcium nitrate	100	100	2.5			4.7							
	Coating potassium	100	100	0.4		7.7								
	Long magnesium sulfate	100	100					1.1						
	Total			5.0	0.8	9.8	4.7	1.2	16.0	8.0	1.2	4.0	4.8	1.6
CRF-H	Eco long total 313	100	70	1.6	0.6	1.6		0.2	24.0	12.0	1.8	6.0	7.2	2.4
	Super eco long	s100	s70	1.7	0.6	1.7								
	Long calcium nitrate	100	100	3.7			7.1							
	Coating potassium	100	100	0.6		11.5								
	Long magnesium sulfate	100	100					1.6						
	Total			7.5	1.2	14.8	7.1	1.8	24.0	12.0	1.8	6.0	7.2	2.4
LF (mg/L)^e^				130	26	168	82	18	1.2	0.3	0.02	0.01	0.1	0.01

a Amount of supplied fertilizer in CRF-L, -M, -H and nutrient concentration in supplied nutrient solution of LF are shown respectively.

b Number of days until 80% of the amount is released at 25°C.

c This fertilizer contained N:P_2_O_5_:K_2_O:MgO in the ratio by weight of 13:11:13:2, plus small amounts of micronutrients.

d Nutrients are released sigmoidally in contrast to the linear nutrient release in other fertilizers.

e Composition at 1.4 dS·m^−1^ EC.

In each fertilizer treatment, the plant density was varied by changing the spacing between plants (20 cm or 30 cm) in each block: 3.70 or 5.56 plants•m^−2^. The experiment was a split-plot design with 3 replicates, with the plant densities set as sub-factors, and fertilizer treatments as the main factor. One 3-m long bed was used for the main plot (fertilizer) and each plot was divided into 2 sub-plots (plant density) containing 15 plants. Each plot was considered a replicate block and each block was placed in a separate row.

### Measurements

The EC and total inorganic nitrogen concentrations (NO_3_-N+NH_4_-N) of nutrient solution in the circulating tank were measured twice a week at 09:00 using an EC meter (B-173; HORIBA, Ltd., Kyoto, Japan), and once with ion chromatography (DX-AQ; Nippon Dionex K.K., Osaka, Japan), respectively. The operating parameters of the ion chromatography were as follows: analytical column AS12A (4 mm) with guard column AG12A (4 mm); a solution with 2.7 mM Na_2_CO_3_ and 0.3 mM NaHCO_3_ as eluent; 1.2 mL·min^−1^ eluent flow rate; injection volume of 0.1 mL. The quantification was obtained through conductivity measurements. The EC and inorganic nitrogen concentrations in the substrate solution were also measured once a week using the same equipment.

Individual mature fruits from 6 plants were harvested from each plot once or twice a week, and the fresh weight of each fruit was measured. Marketable fruit was defined as fruit weighing more than 80 g with no physiological damage such as blossom-end rot. The leaf area (LA) of 6 plants from each plot was destructively measured at the end of each experiment using an area meter (AAM-7, Hayashi Denko, Tokyo, Japan). Any dead leaves of the lower parts of the plants were dried in an open-air oven at 80°C over 3 days; the LA was estimated from the ratio of LA to the dry weight of live leaves. The sum of LA of both live and dead leaves was regarded as the LA just after pinching. Remaining CRF after cultivation was dried in an open-air oven at 80°C for 1 week and total nitrogen content of the dried CRF was measured using the NC analyzer (Vario MAX CN; Elementar Analysensysteme, Germany).

The air and water temperatures in the circulation tank were measured using thermocouples and the averages were recorded every 10 minutes by a data logger (ZR-RX40V; OMRON Corp., Kyoto, Japan). Daily nitrogen release from the CRF was calculated based on the water temperature in the circulation tank, using simulation software (Sehi-meijin ver. 2.0; JA Zen-noh, Tokyo, Japan). The daily amounts of irrigation and drainage were also recorded.

Light interception by the plant canopy was measured using simple recording film (Opt leaf R-2D; Taisei E&L, Tokyo, Japan) to measure cumulative solar radiation according to previous methods [15]–[18] during the periods of June 8−13, 2012 (SS), September 12−16, 2012 (SF), and January 16−27, 2013 (FW). The film measured cumulative solar radiation through its gradations of fading after direct exposure to radiation at the same time at many points. Nine films (18 mm ×35 mm) were laid on rectangular planks (18 mm width ×1820 mm length), which were placed vertically on the planting beds to measure light interception by the plant canopy as described by Shiraiwa et al. [19]. The integrated solar radiation above the plant canopy in the greenhouse was also determined using the film and a pyranometer (LI-200SA; Li-Cor, Lincoln, USA). The following regression equation was developed using the degrees of fading of the films and the amount of radiation determined by the pyranometer to estimate the amount of intercepted radiation: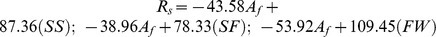; where *R_s_* is cumulative solar radiation (MJ•m^−2^) and *A_f_* is the light absorbance of the film.

### Data analysis

Regression analysis, analysis of covariance (ANCOVA) and Tukey's multiple comparison tests were performed using the SAS Add-In for Microsoft Office 5.1 (SAS Institute Inc., Cary, NC, USA).

## Results and Discussion

### Climatic conditions during the experiment

**Figure 2:**
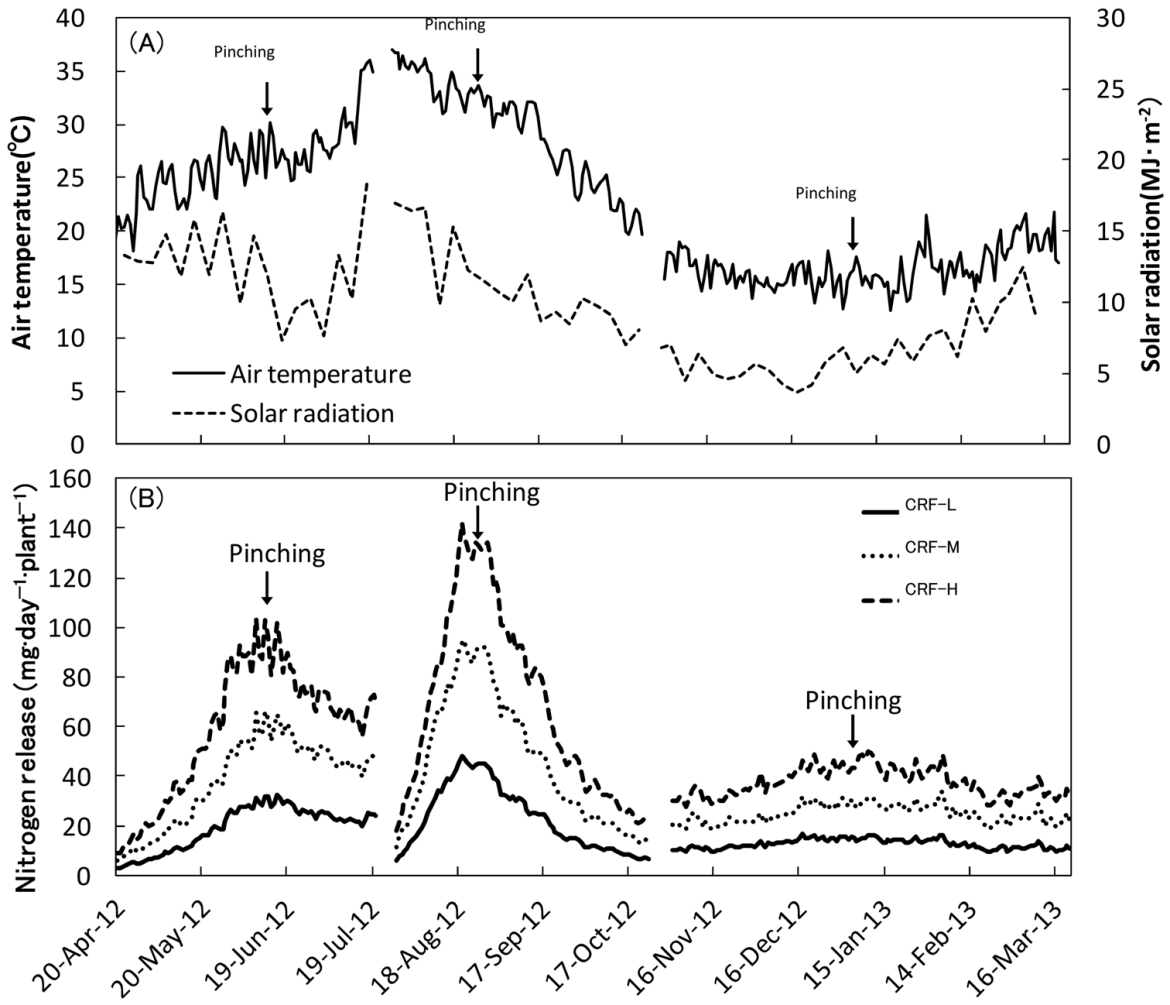
Mean daily air temperature and 5-day solar radiation inside the greenhouse (A) and daily nitrogen release rate from CRF (B) during each cropping season.

Climatic conditions during the experiment are shown in Figure 2A. The mean air temperatures for the entire experimental period were 16.4°C (SS), 29.5°C (SF) and 16.5°C (FW). The mean 5-day solar radiation for the entire experimental period was 16.4 MJ·m^−2^·day^−1^ (SS), 16.5 MJ·m^−2^·day^−1^ (SF) and 10.2 MJ·m^−2^·day^−1^ (FW). They were highest at the end period of SS and the beginning of SF (approximately 35°C and 20 MJ·m^−2^·day^−1^) and lowest during the first part of FW (approximately 15°C and 5 MJ·m^−2^·day^−1^). Calculated daily amount of nitrogen released from CRF is shown in Figure 2B. It increased until the pinching period, and then decreased gradually until the end of the experiment in each growing season, although levels were dependent on the cropping season; the average values of fertilizer levels during the experimental were 19.3−59.2 mg·day^−1^·plant^−1^ (SS), 23.0−68.3 mg·day^−1^·plant^−1^ (SF) and 12.6−37.4 mg·day^−1^·plant^−1^ (FW). Nitrogen release levels were positively correlated with CRF supply. Amounts of total nitrogen released from CRF during the experiment are shown in Table 2. The actual values were larger by 6−9% in SS, and smaller by 1−7% and 10−13% in SF and FW, respectively, than each calculated value. Thus, these were comparable to the calculated values in SS and SF, while, the value was lower in FW than in the other seasons. It should be noted that the calculated values in Fig. 2B may be slightly different from the actual values.

**Table 2:** Amount of nitrogen released from CRF in each cropping season.

Cropping season	Fertilizer rate	Amount of nitrogen released from CRF (g•plant^−1^)		A/B
		Actual value (A)	Calculated value (B)	
SS	CRF-L	1.88	1.77	1.06
	CRF-M	3.84	3.53	1.09
	CRF-H	5.82	5.44	1.07
SF	CRF-L	1.94	2.07	0.94
	CRF-M	3.85	4.13	0.93
	CRF-H	6.09	6.15	0.99
FW	CRF-L	1.60	1.77	0.90
	CRF-M	3.04	3.49	0.87
	CRF-H	4.58	5.27	0.87

### Nutrient concentration in the circulation tank and substrate solution

**Figure 3:**
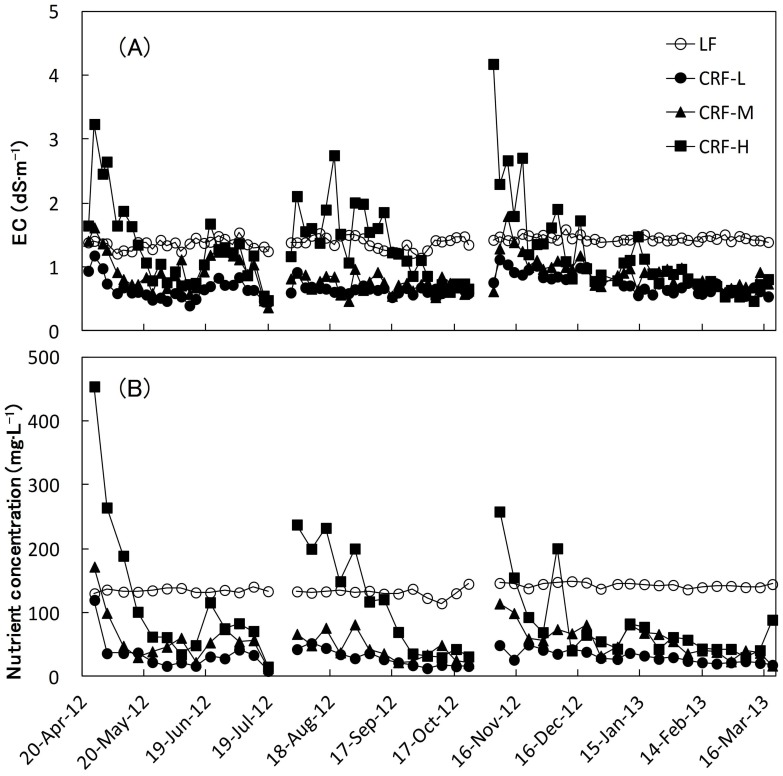
EC (A) and nutrient concentration (nitrogen equivalent) (B) in the circulating tank during each cropping season.

**Figure 4:**
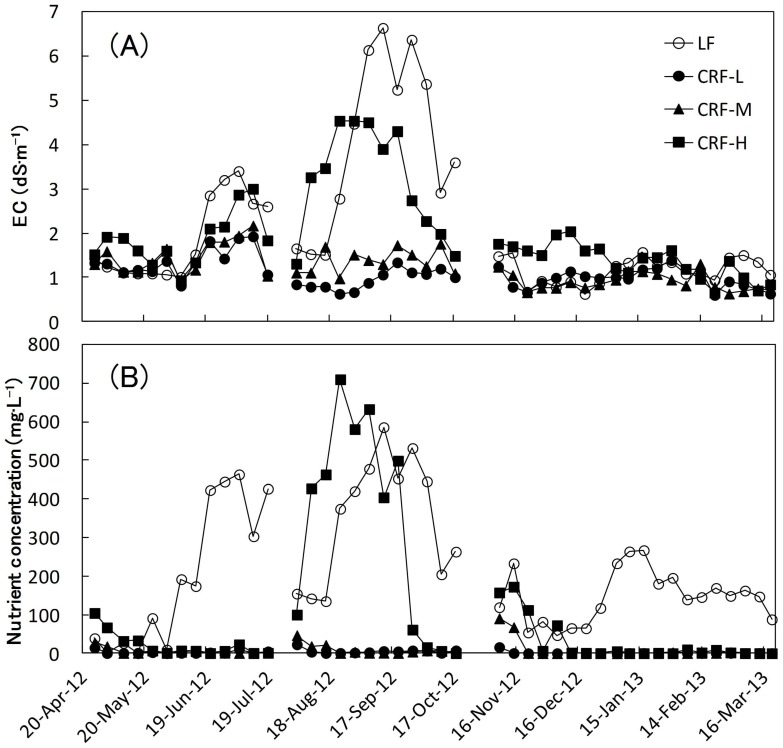
EC (A) and nutrient concentration (nitrogen equivalent) (B) in the substrate solution with each cropping season.

The EC and the nutrient concentration (nitrogen equivalent) in the circulation tank and substrate solution during the experiment are shown in Figures 3 and 4, respectively. During the experiment, EC and nitrogen concentration in CRF-L and CRF-M were almost always lower than those in the LF treatment, for which the EC value was approximately 1.4 dS•m^−1^ and N concentration was approximately 130 mg·L^−1^. However, those in CRF-H were higher than those in the LF treatment during the first part of the experiment in each cropping season. Between SS and FW treatments, the substrate solution showed a large difference in EC values, while EC in CRF-H and LF treatments were much higher than for CRF-S and CRF-M groups. The nutrient concentration was lower in CRF-L and CRF-M than in the LF treatment and the value was generally extremely low (nearly 0 mg·L^−1^), except for the beginning of the experiment in each growing season. On the other hand, the value for CRF-H was similar to other CRF treatments in SS, and LF treatment in SF and FW. However, no plants were identified as having resulting physiological damage except for a few fruits with physiological disorders that were seen in all treatments. Therefore, there was no concern about the extreme accumulation of inorganic ions in the substrate solution in the CRF treatments (closed system), a situation that approximated the LF treatment (open system).

### Relationship between leaf area index (LAI) and nutrient supply

**Figure 5:**
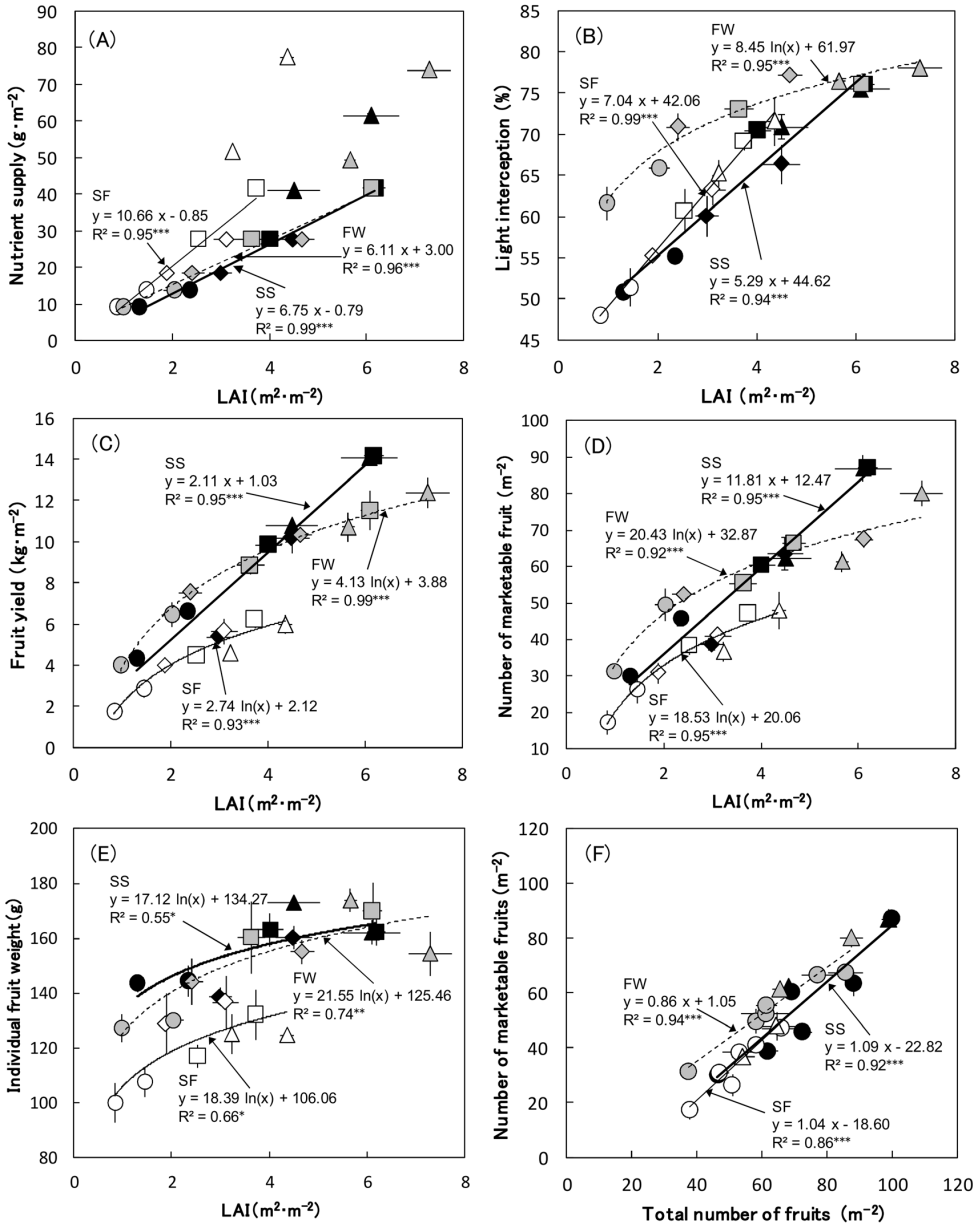
The relationship between leaf area index (LAI) and the various agronomic traits. The total amount of the nutrient supply (N equivalent) (A), light interception by plants (B), marketable fruit yield (C), number of marketable fruit (D), individual fruit weight (E), and the relationship between the total number of fruits and the number of marketable fruits (F). Symbols and regression lines represent the following: (1) Fertilizers, LF: triangle; CRF-L: circle; CRF-M: diamond; CRF-H: square; (2) Cropping seasons, SS: filled; SF: open; FW: gray; (3) regression lines, SS: thick; SF: thin; FW: dotted. Vertical and horizontal bars represent standard errors of the means (n = 3). *P<0.05; **P<0.01; *** *P*<0.001.

Nutrient supply (N equivalent) was higher in LF treatments than in all CRF treatments in each cropping season (Table 3). Figure 5A shows the relationship between the amount of nutrient supplied on a ground area basis during the experiment and LAI at pinching. In CRF treatments, the nutrient supply linearly increased with increasing LAI, irrespective of fertilizer rate and plant density in each cropping season. When compared to the treatments with similar LAI, CRF treatments supplied a significantly lower level of nutrients per ground area than LF treatments in each cropping season, according to the ANCOVA analysis (*P* = 0.012−0.031). The following can be understood from these results: (1) controlling LAI through nutrient management using CRF is probably possible, (2) the amount of nutrient supply can be reduced further using CRF instead of LF. On the other hand, among the 3 cropping seasons, larger amounts of nutrients were required in SF than in the others to achieve the same LAI; the ANCOVA indicated that slope in the line of SF was significantly larger than that in other lines (*P*<0.01).

**Table 3:** Nutrient supply (N equivalent), leaf area index (LAI) and light interception by the plant canopy, and cumulative fruit yield at each treatment.

Cropping season	Treatment		Nutrient supply (g•m^−2^)	LAI (m^2^•m^−2^)	Light interception (%)	Fruit yield (kg•m^−2^)	
	Plant density	Fertilizer				Total	Marketable
Spring-summer	Low	LF	41.1	4.5 ab	70.9ab	11.5b	10.8b
		CRF-L	9.3	1.3 d	50.8e	5.1d	4.3d
		CRF-M	18.5	3.0 bcd	60.1cd	6.5cd	5.4cd
		CRF-H	27.8	4.0 bc	70.5ab	10.5b	9.9b
	High	LF	61.6	6.1 a	75.5a	15.5a	14.1a
		CRF-L	13.9	2.3 cd	55.2de	8.0c	6.6c
		CRF-M	27.8	4.5 ab	66.4bc	11.4b	10.2b
		CRF-H	41.7	6.2 a	76.1a	14.9a	14.2a
Summer-fall	Low	LF	51.7	3.2 b	65.3abc	5.4abc	4.6abc
		CRF-L	9.3	0.8e	48.0e	2.8d	1.8d
		CRF-M	18.5	1.9d	55.3de	4.7c	4.0bc
		CRF-H	27.8	2.5c	60.7cd	5.1bc	4.5abc
	High	LF	77.6	4.4a	71.7a	6.9ab	6.0a
		CRF-L	13.9	1.4de	51.4e	4.1cd	2.9cd
		CRF-M	27.8	3.1b	63.3bc	6.4ab	5.7ab
		CRF-H	41.7	3.7ab	69.2ab	7.1a	6.3a
Fall-winter	Low	LF	49.4	5.6bc	76.5ab	11.2ab	10.7a
		CRF-L	9.3	1.0f	61.7e	4.4d	4.0d
		CRF-M	18.5	2.4e	70.9cd	8.0c	7.5c
		CRF-H	27.8	3.6cd	73.1bc	9.5bc	8.9bc
	High	LF	74.1	7.3a	78.1a	13.1a	12.4a
		CRF-L	13.9	2.0ef	65.9d	7.2c	6.5cd
		CRF-M	27.8	4.7c	77.2ab	10.9ab	10.3ab
		CRF-H	41.7	6.1b	76.1ab	12.8a	11.5a

The same letter after numerical values in the same cropping season and within the same column indicates no significant difference at *P*<0.05.

### Relationship between LAI and light interception by the plant canopy

The difference in light interception by the plant canopy at the pinching period between treatments showed a similar trend to that of LAI, but the difference between treatments was smaller, particularly for FW (Table 3). Figure 5B represents the relationship between LAI and light interception. The light interception showed a corresponding increase with LAI to approximately 6 m^2^·m^−2^, although the saturation point was not observed in SS or SF for any treatment; the others maximized at approximately 4 m^2^·m^−2^. The light interception at lower LAIs was higher in FW than in SS and SF. Thus, the relationship between LAI and light interception was different among the cropping seasons. The main reason for the difference in the relationship between LAI and light interception is probably the difference in solar radiation throughout the seasons. It has been reported that light interception reflects diurnal and seasonal differences in various plants, resulting from changing solar elevation [20]–[24]. In a tomato canopy, light penetration has also been found to be stronger in summer than in winter [25], increasing with solar elevation. This phenomenon provides an explanation for the higher level of light interception in FW at a lower LAI: solar elevation was lower in FW than in SS and SF. These results indicate that an LAI higher than 4 m^2^·m^−2^ may not be necessary to increase light interception in a fall-winter cropping season. On the other hand, the highest value of light interception in this study was 75−80%, even when LAI was very high (over 6 m^2^·m^−2^). Heuvelink et al. [26] reported that the light interception was approximately 90% when the LAI was 3.5 m^2^·m^−2^ inside greenhouses of Dutch tomato growers equipped with a high wiring system. These facts indicate that more light was intercepted by plants inside the Dutch tomato greenhouses than inside the greenhouse in this study. This may be because our ratio of plant height to distance between beds was larger than that of the Dutch study.

### Relationship between LAI and the fruit yield

In general, larger total and marketable fruit yields corresponded with higher fertilizer rate and plant density among CRF treatments in each cropping season, and did not differ significantly between LF and CRF-H for either plant density except in the FW and low plant density group (Table 3). Since there was a very strong linear correlation between total and marketable fruit yields in each cropping season (R^2^ = 0.987−0.993, *P*<0.001), the value of marketable yield was applied in the following regression analysis. The yields increased linearly with increasing LAI to approximately 6 m^2^·m^−2^ and the saturating point was not observed in SS for any treatments; however, the yields reached the maximum at approximately 3 to 4 m^2^·m^−2^ in SS and FW (Figure 5C). The relationship between light interception and fruit yield is generally strong because there is a linear relationship between fruit yield and solar radiation incident on tomato crops [12]. In the case of SS and FW, the relationship between LAI and light interception corresponded strongly with that of LAI and the fruit yield. Therefore, the difference in light interception among the treatments probably resulted in the fruit yield in SF and FW. From these results, we find that maximizing LAI to the saturation point of light interception is probably important to improve the fruit yield in spring-summer and fall-winter seasons.

In the case of SF, on the other hand, the fruit yield was maximized at an LAI of approximately 3 m^2^·m^−2^, although light interception linearly increased to an LAI of 4.5 m^2^·m^−2^. Therefore, increasing light interception by expanding LAI did not contribute to an improvement in fruit yield in SF as it did during the other cropping seasons. The marketable fruit yield per unit area can be divided into 2 components: the number of marketable fruit per unit area and the average fresh fruit weight per individual marketable fruit. The number of marketable fruit in SF maximized at approximately 3 m^2^·m^−2^ of LAI, which was smaller than in the other cropping seasons at similar LAI (Figure 5D). The individual fresh weight of marketable fruit also maximized at approximately 3 m^2^·m^−2^ of LAI in SF (Figure 5E). These results indicate that both the number of marketable fruit and the individual fruit weight did not increase along with increasing LAI and the light interception in SF, as in the other cropping seasons. The total number of fruit per unit area was strongly correlated with the number of marketable fruit (Figure 5F). The slope of the regression line for SF was not significantly lower than those for SS and FW, according to the ANCOVA analysis. Hence, a lower number of marketable fruit in SF was mainly a result of the lower total number of fruit. In general, the number of fruit decreases during the summer season in Japan because of high temperatures [27], [28]. It has been reported that the rate of fruit set decreases when the average air temperature is over 25°C [29], [30]. On the other hand, high temperature at reproductive stages also decreases individual fresh fruit weight because of a reduction in numbers of fruit cells and seeds [30], [31]. In SF, the daily average temperatures during the reproductive stages (before pinching) were over 30°C. Therefore, the lower number of fruit and lower individual fresh fruit weights in SF may be caused by high temperature at reproductive stages, indicating the necessity of increasing fruit yield by improving these traits during the summer cropping season in Japan.

From these results, we concluded that the optimal LAI may differ depending on the cropping seasons, corroborating the results of Hosoi [11] in which the optimal LAI for tomato fruit yield differs depending on season. The optimal LAI at pinching may be 6 m^2^•m^−2^ in SS, 3 m^2^•m^−2^ in SF, and 4 m^2^•m^−2^ in FW, judging from the fruit yield and nutrient use efficiency results. In the case of the LAI values with similar fruit yields (CRF-H and LF at high density in SS; CRF-M at high density and LF at low density in SF and FW), the nutrient supply was 32% (SS), 46% (SF) and 44% (FW) lower in CRF systems compared to that in LF systems.

On the other hand, the optimal LAI for light interception and dry matter production is different depending on the light extinction coefficient (LEC) [32]. Higashide and Heuvelink [33] reported that the LEC differs among tomato varieties. Therefore, the optimal LAI may differ depending on not only the cropping season but also the cultivars used. Further research into this aspect is needed.

## Conclusions

Application of CRF to a water-circulating tank in a closed system enables growers to achieve a desirable LAI by adjusting the amount of nutrient supply per unit area. Fertilization with CRF also greatly reduces nutrient use without decreasing fruit yield, with LAI values similar to the open LF system with EC-based management. However, it may be necessary to increase light interception by the plant canopy by improving the planting system, particularly in a tomato cultivation system with a low node-order pinching and high plant density.
